# Impact of the COVID-19 pandemic on racial disparities in adolescent bariatric surgery: an MBSAQIP analysis

**DOI:** 10.1007/s00464-026-12955-7

**Published:** 2026-06-16

**Authors:** Nikita S. Thareja, Ana Garcia Cabrera, J. Jeffery Reeves, Hannah M. Hollandsworth, Bryan J. Sandler, Garth R. Jacobsen, Ryan C. Broderick

**Affiliations:** Division of Minimally Invasive Surgery, University of California, San Diego (UCSD), 9300 Campus Point Drive, La Jolla, CA 92037 USA

**Keywords:** Adolescent bariatric surgery, Racial disparities, COVID-19, MBSAQIP, Health equity, Metabolic surgery

## Abstract

**Background:**

Metabolic and bariatric surgery (MBS) is the most effective intervention for severe adolescent obesity, yet persistent disparities exist in surgical access. The COVID-19 pandemic disrupted elective surgery and may have altered access for historically underserved populations.

**Methods:**

Using MBSAQIP, we identified adolescents aged 19 years or younger who underwent MBS from 2015 to 2023, stratified into three phases: pre-COVID (2015–2019), acute COVID (2020–2021), and recovery (2022–2023). Race/ethnicity was classified using Hispanic-priority methodology. Racial and ethnic composition, comorbidity burden, and 30-day outcomes were compared using Fisher’s exact and Kruskal–Wallis tests. Firth penalized logistic regression was used for sensitivity analysis of rare outcomes.

**Results:**

Overall, 5432 adolescents underwent MBS from 2015 to 2023. White representation declined from 49% pre-COVID to 32% during recovery (− 17%; 95% CI − 19%, − 14.7%), while Hispanic representation increased from 25 to 36% (+ 11%; 95% CI + 9.1%, + 13.2%) and Black representation from 17 to 21% (+ 4%; 95% CI + 2.6%, + 6.2%); cohort composition differed significantly across phases (*p* < 0.001). Comorbidity burden was stable. Overall, 30-day complication rates were low (1.0%). Among Black patients, complication rates declined from 3.9% pre-COVID to 0.8% during recovery (− 3.1%; 95% CI − 5.7%, − 2.1%; *p* = 0.004). In sensitivity analysis, Black patients had higher odds of complications compared with White patients (OR 3.21; 95% CI 1.55, 6.71; *p* = 0.002). No individual complication type differed significantly after Benjamini–Hochberg correction. Readmission and reoperation rates did not differ significantly across phases.

**Conclusions:**

The racial and ethnic composition of adolescents undergoing MBS shifted substantially across the COVID-19 pandemic, with increased representation of historically underserved racial and ethnic groups. Complication disparities observed among Black patients pre-COVID were not statistically significant in later phases, though sparse event counts limit interpretation. Prospective studies are needed to clarify the mechanisms driving pandemic-era changes in equity of access to adolescent MBS.

**Graphical Abstract:**

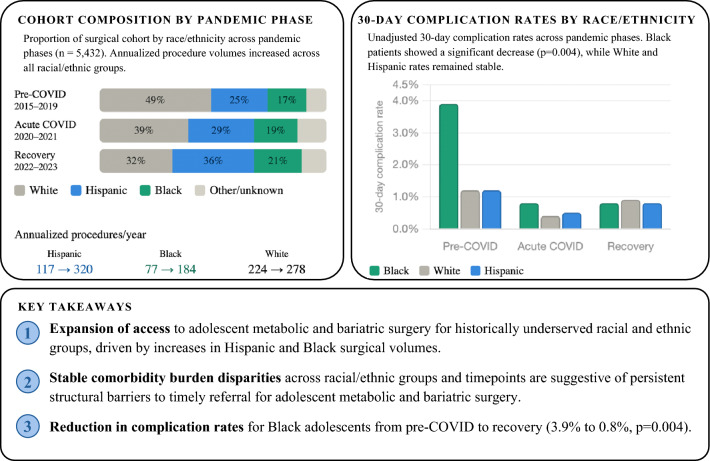

**Supplementary Information:**

The online version contains supplementary material available at 10.1007/s00464-026-12955-7.

Severe obesity in adolescents is a critical public health challenge in the United States, with prevalence rising steadily over the past two decades [1–3]. Current estimates suggest that approximately 21% of adolescents meet criteria for obesity, placing them at elevated risk for type 2 diabetes, hypertension, cardiovascular disease, and other long-term health consequences [2–4]. Metabolic and bariatric surgery (MBS) is increasingly recognized as the most effective and durable intervention for adolescents with severe obesity, producing substantial and sustained weight loss alongside marked improvements in obesity-related comorbidities [2, 3, 5, 6]. Despite its demonstrated efficacy, MBS remains critically underutilized, with fewer than 0.1% of eligible adolescents receiving surgery, highlighting a substantial treatment gap [7–9]. Barriers to access include limited referrals from primary care, insurance restrictions, geographic disparities in surgeon availability, and social factors such as caregiver and transportation availability [9–13]. Clinical guidelines from professional societies, including the American Society for Metabolic and Bariatric Surgery, the American Academy of Pediatrics, and the Endocrine Society, now recommend MBS for appropriately selected adolescent patients, reflecting a growing consensus regarding its safety and efficacy [13–15].

Despite these advances, pronounced racial and ethnic disparities exist in access to bariatric surgery [3]. Black and Hispanic adolescents undergo MBS at substantially lower rates than White adolescents, despite experiencing disproportionately high rates of severe obesity [7, 16–18]. Adolescents from historically underserved racial and ethnic groups also often present for surgical evaluation with higher body mass indices and greater burdens of obesity-related comorbidities, highlighting inequities in preoperative access, referral patterns, and timing of intervention [17–19]. Importantly, postoperative outcomes and recovery times are largely comparable across racial groups, suggesting that disparities in bariatric care arise primarily from inequities in access to surgical treatment rather than differences in the quality of surgical care [18–21].

Understanding these disparities requires context regarding the structure of the United States healthcare system. Unlike countries with universal healthcare coverage, the United States relies on a fragmented system in which most individuals obtain coverage through employer-sponsored private insurance, government-sponsored programs for low-income (Medicaid) and elderly patients (Medicare), or individually purchased plans [22–24]. Access to bariatric surgery is influenced heavily by insurance type and individual payer criteria, which vary by insurer and state [25]. Many private insurers require documentation of prior supervised weight-management attempts, specific body mass index thresholds, comorbidity documentation, and pre-authorization before approving coverage, which can create administrative barriers that disproportionately affect patients with limited insurance coverage or fragmented primary care relationships [26–28]. Adolescent bariatric surgery has additional access constraints because pediatric-accredited bariatric programs are geographically concentrated in academic medical centers, and not all insurance payers cover adolescent procedures [9, 12]. Race and ethnicity intersect with these structural factors through well-documented disparities in insurance coverage, income, geographic proximity to accredited centers, and primary care referral rates, which can produce persistent gaps in surgical access [17, 29, 30].

Against this backdrop of existing structural inequities, the COVID-19 pandemic profoundly disrupted healthcare delivery worldwide. Elective surgical procedures were postponed or canceled during the early phases of the pandemic, and healthcare systems faced unprecedented challenges related to staffing shortages, resource constraints, and changes in care delivery[31, 32]. These disruptions may have altered referral patterns, surgical prioritization, and access to specialty care. Simultaneously, the rapid expansion of telemedicine and remote patient evaluation created new avenues for care delivery that may have mitigated access barriers for historically underserved populations. While prior studies have examined the impact of COVID-19 on overall surgical volume and bariatric surgery utilization, little is known about how the pandemic influenced racial disparities in adolescent bariatric surgery rates [16, 33–35]. Evaluating how pandemic-era disruptions influenced both access to surgery and postoperative outcomes can provide important insights into modifiable systemic factors that contribute to disparities in surgical care.

Using the MBSAQIP registry, a large multicenter database of prospectively collected surgical outcomes [36], we examined the following question: among adolescents with severe obesity undergoing bariatric surgery at MBSAQIP-accredited centers, did exposure to the COVID-19 pandemic alter the racial and ethnic composition of the surgical cohort and 30-day postoperative complication rates compared with the pre-pandemic period?

## Materials and methods

### Data source and study population

We performed a retrospective cohort analysis of the Metabolic and Bariatric Surgery Accreditation and Quality Improvement Program (MBSAQIP) Participant Use Files, which contain prospectively collected, de-identified data from accredited bariatric surgery centers in North America. The MBSAQIP database includes detailed information on patient demographics, comorbid conditions, perioperative variables, and 30-day postoperative outcomes. This study utilized a de-identified, publicly available dataset and was therefore exempt from institutional review board oversight in accordance with federal regulations governing human subjects research (45 CFR 46.102). As the dataset is fully de-identified, individual participant informed consent was not required. This study was reported in accordance with the Strengthening the Reporting of Observational Studies in Epidemiology (STROBE) guidelines.

Patients aged 19 years or younger who underwent bariatric surgery between 2015 and 2023 were included in the study. Variables with partially missing data were analyzed using available cases; missing data are reported where present. The study population was divided into three time periods to reflect phases of the COVID-19 pandemic: pre-COVID (2015–2019), acute COVID (2020–2021), and recovery (2022–2023). Global data have demonstrated a substantial disruption to bariatric surgery during 2020, with partial but incomplete recovery in 2021, indicating a distinct acute pandemic phase [37]. Accordingly, we defined 2020–2021 as the acute COVID period and 2022–2023 as a recovery phase. These phase definitions represent one reasonable categorization of the pandemic timeline; alternative definitions may yield different results, and sensitivity analyses with alternative boundaries were not performed.

### Race/ethnicity classification

A combined race/ethnicity variable was constructed for all analyses. Patients identifying as Hispanic were classified as Hispanic regardless of reported race, consistent with federal standards for race and ethnicity reporting [38, 39]. The remaining patients were classified by race as White, Black, Asian, Other, or Unknown to represent non-Hispanic patients within those groups. Due to small sample sizes, patients identified as some other race (*n* = 52), American Indian or Alaska Native (*n* = 10), Native Hawaiian or Pacific Islander (*n* = 7), and multiracial combinations (*n* = 15) were classified as Other.

### Clinical variables

Demographic variables included age, sex, and the combined race/ethnicity variable described above. Clinical covariates included diabetes mellitus, hypertension, obstructive sleep apnea, hyperlipidemia, GERD, smoking status, history of prior surgery, and ASA classification. Diabetes status was harmonized across MBSAQIP coding schema changes in 2020 and classified as present (insulin-dependent or non-insulin-dependent) or absent. Hypertension was classified as a binary variable (any antihypertensive medication use versus none); due to a coding inconsistency in the 2015 MBSAQIP dataset in which the “no antihypertensive” response option was absent, hypertension data from 2015 are reported as missing and excluded from hypertension analyses. Operative variables included the procedure performed and the operative duration; procedure type (initial versus revisional) and surgical approach were excluded from comparative analyses due to inconsistent variable coding across MBSAQIP data collection years.

### Outcomes

The primary outcome was the occurrence of any postoperative complication within 30 days of surgery. A composite complication variable was created from 17 MBSAQIP-defined postoperative complications, including surgical site infections, anastomotic leak, wound disruption, pneumonia, unplanned intubation, pulmonary embolism, ventilator dependence, renal insufficiency, urinary tract infection, cerebrovascular events, cardiac arrest, myocardial infarction, thrombosis, sepsis, and septic shock. Secondary outcomes included 30-day readmission, reoperation, and length of stay. Thirty-day mortality was not analyzed due to inconsistent variable coding in the MBSAQIP dataset after 2019.

### Statistical analysis

As this study represents a retrospective analysis of all eligible adolescents captured in the MBSAQIP Participant Use Files between 2015 and 2023, no sample size or statistical power calculation was performed; the full available cohort was included. Descriptive statistics were used to summarize patient demographics and clinical characteristics across COVID-19 phases. Continuous variables were assessed for normality using the Shapiro–Wilk test and visual inspection of histograms and Q–Q plots. All variables demonstrated non-normal distributions, with substantial right skew observed for length of stay (skewness 3.7–12.2) and operative time (skewness 2.0–4.4). Continuous variables are therefore reported as medians with interquartile ranges and compared across groups using the Kruskal–Wallis test. Categorical variables were summarized as counts and percentages and compared using Fisher’s exact test with simulated *p* values (10,000 iterations) to accommodate sparse cells. A global random seed was set prior to analysis to ensure full reproducibility of all Monte Carlo simulations. Wilson confidence intervals were calculated for racial and ethnic proportions within each pandemic phase.

Comorbidity burden was summarized and compared descriptively across pandemic phases within White, Black, and Hispanic patients. Asian (*n* = 50), Other (*n* = 84), and Unknown (*n* = 463) groups were excluded from phase-level comorbidity comparisons due to small sample sizes that precluded meaningful stratified analysis. Given that the MBSAQIP dataset captures only patients who underwent surgery, these findings reflect the characteristics of the surgical cohort and cannot be used to infer changes in population-level disease burden or referral patterns.

Across COVID-19 phases, unadjusted complication rates were compared within each racial and ethnic group using Fisher’s exact test, and length of stay was compared using the Kruskal–Wallis test. Multivariable regression was not performed because several race-phase strata had sparse complication events, resulting in fewer than 10 events per variable in critical groups (Supplemental Fig. 1). To avoid model overfitting and unreliable odds ratio estimates, analysis was therefore limited to unadjusted comparisons, as discussed further in the limitations section. For individual complication comparisons, Benjamini–Hochberg false discovery rate correction was applied to account for multiple comparisons.

**Fig. 1:**
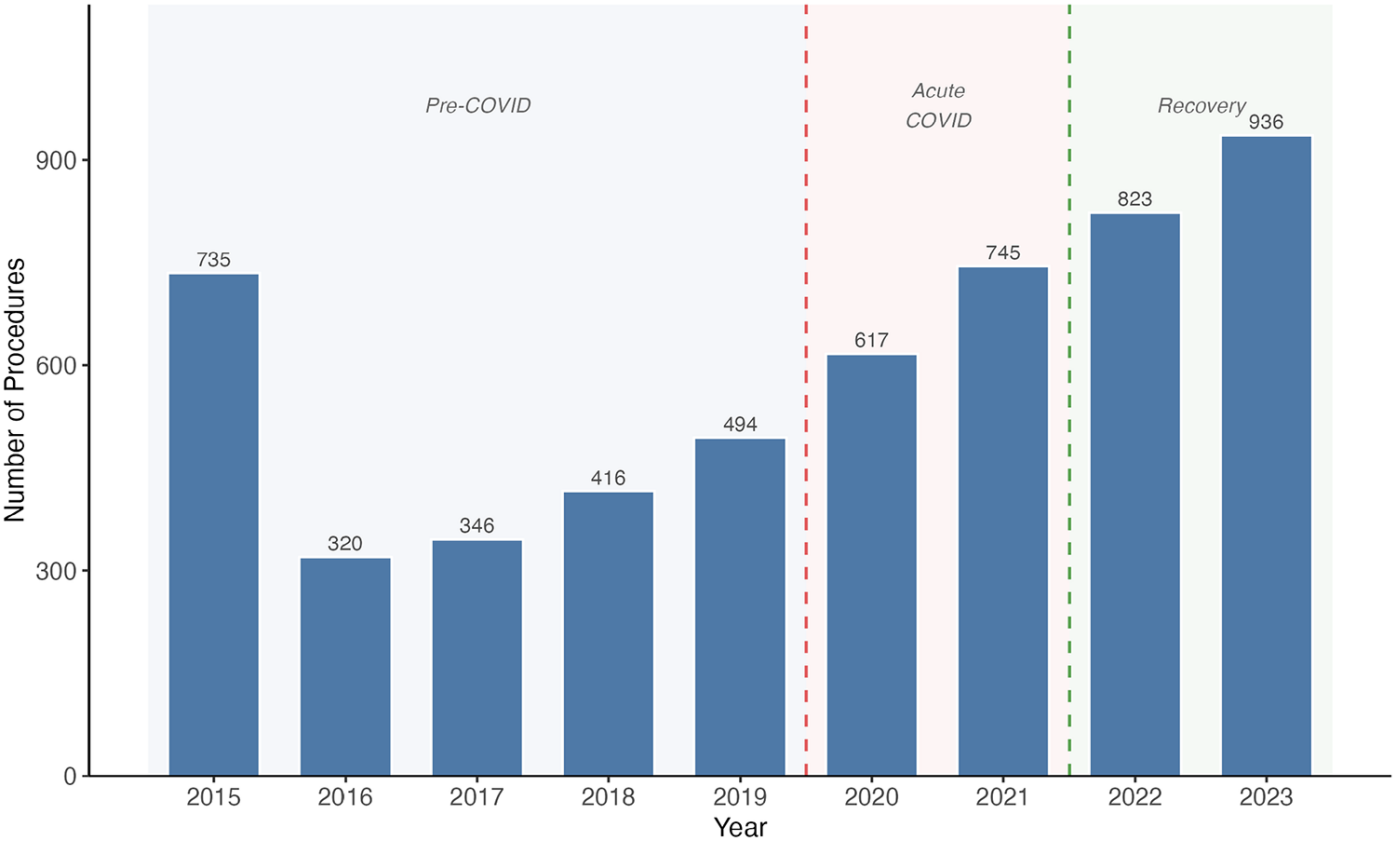
Annual volume of adolescent metabolic and bariatric surgery at MBSAQIP-accredited centers, 2015–2023. Shaded regions denote pandemic phases; dashed vertical lines indicate phase boundaries. Numbers above bars indicate annual procedure counts

As a sensitivity analysis, Firth penalized logistic regression was used to evaluate the association between race/ethnicity and complication risk while accounting for the sparse event counts that precluded standard logistic regression. Models were fit with and without race × COVID phase interaction terms, and a fully adjusted model was constructed, including age, BMI, sex, diabetes, hypertension, obstructive sleep apnea, ASA classification, and procedure category. Firth penalized regression applies a likelihood penalty that produces reliable estimates in the setting of rare events and complete or quasi-complete separation.

All analyses were performed by Nikita Thareja, MD, using R statistical software (R version 4.4.0; R Foundation for Statistical Computing, Vienna, Austria). Firth penalized logistic regression was performed using the logistf package (version 1.26) in R. Statistical significance was defined as a two-sided *p* value < 0.05.

## Results

### Surgical volume

A total of 5,432 adolescent bariatric procedures were performed at MBSAQIP-accredited centers between 2015 and 2023. After an initial decrease from 2015 to 2016, annual surgical volume increased steadily through 2023, rising from 320 procedures in 2016 to 936 in 2023. Volume continued to increase through the acute COVID phase (2020: *n* = 617; 2021: *n* = 745) and recovery period (2022: *n* = 823; 2023: *n* = 936). The absence of a measurable volume decline during 2020–2021 may reflect prioritization of adolescent bariatric surgery as a semi-urgent metabolic intervention during the pandemic, or limitations of annual procedure counts in capturing short-term disruptions, with transient volume loss potentially offset by subsequent catch-up within the same calendar year (Fig. 1).

### Patient characteristics

Patient demographics and clinical characteristics are summarized in Table 1. The cohort was predominantly female (72%) with a median age of 17 years (IQR 16–18) and a median BMI of 46 kg/m^2^ (IQR 41–52). The proportion of male patients increased from 25% in the pre-COVID period to 32% during recovery (*p* < 0.001). ASA classification shifted toward greater comorbidity burden over time, with ASA III–IV patients comprising 65% of the pre-COVID cohort and 73% of the recovery cohort (*p* < 0.001). The prevalence of GERD declined from 13 to 9.0% (*p* = 0.001) and active smoking from 2.8 to 1.1% (*p* < 0.001), while hyperlipidemia increased modestly from 2.0 to 3.5% (*p* = 0.005). Most other comorbidities, including diabetes, hypertension, and obstructive sleep apnea, did not differ significantly across phases.

**Table 1:** Patient demographics, comorbidities, and operative characteristics by pandemic phase

Characteristic	Overall *N* = 5432	Pre-COVID *N* = 2311	COVID *N* = 1362	Recovery *N* = 1759	*p* value
Age, years (median, IQR)	17.0 16.0, 18.0)	17.2 (16.2, 17.9)	17.0 (16.0, 18.0)	17.0 (16.0, 18.0)	** < 0.001**
BMI, kg/m^2^ (median, IQR)	46 (41, 52)	46 (41, 52)	46 (42, 51)	46 (42, 52)	> 0.9
Sex (*n*, %)					** < 0.001**
Female	3907 (72%)	1724 (75%)	990 (73%)	1,193 (68%)	NA
Male	1518 (28%)	587 (25%)	368 (27%)	563 (32%)	NA
Non-binary	7 (0.1%)	0 (0%)	4 (0.3%)	3 (0.2%)	NA
Race/ethnicity (*n*, %)					** < 0.001**
White	2,206 (41%)	1,121 (49%)	530 (39%)	555 (32%)	NA
Black	1009 (19%)	383 (17%)	259 (19%)	367 (21%)	NA
Hispanic	1620 (30%)	586 (25%)	395 (29%)	639 (36%)	NA
Asian	50 (0.9%)	13 (0.6%)	2 (0.1%)	35 (2.0%)	NA
Other	84 (1.5%)	9 (0.4%)	22 (1.6%)	53 (3.0%)	NA
Unknown	463 (8.5%)	199 (8.6%)	154 (11%)	110 (6.3%)	NA
Comorbidities (*n*, %)					
Diabetes	869 (16%)	361 (16%)	204 (15%)	304 (17%)	0.2
Hypertension	316 (6.7%)	118 (7.5%)	74 (5.4%)	124 (7.0%)	0.067
Obstructive sleep apnea	1,178 (22%)	480 (21%)	292 (21%)	406 (23%)	0.2
Hyperlipidemia	153 (2.8%)	46 (2.0%)	45 (3.3%)	62 (3.5%)	**0.004**
GERD	581 (11%)	292 (13%)	130 (9.5%)	159 (9.0%)	**0.001**
Active smoker	120 (2.2%)	64 (2.8%)	36 (2.6%)	20 (1.1%)	**0.001**
History of DVT	10 (0.2%)	5 (0.2%)	4 (0.3%)	1 (< 0.1%)	0.3
History of PE	7 (0.1%)	2 (< 0.1%)	4 (0.3%)	1 (< 0.1%)	0.2
Chronic steroid use	66 (1.2%)	40 (1.7%)	6 (0.4%)	20 (1.1%)	**0.002**
Therapeutic anticoagulation	19 (0.3%)	10 (0.4%)	7 (0.5%)	2 (0.1%)	0.083
Renal insufficiency	6 (0.1%)	2 (< 0.1%)	0 (0%)	4 (0.2%)	0.2
History of prior surgery	43 (0.8%)	30 (1.3%)	5 (0.4%)	8 (0.5%)	**0.001**
ASA classification (*n*, %)					** < 0.001**
ASA I–II	1703 (31%)	797 (35%)	424 (31%)	482 (27%)	NA
ASA III–IV	3717 (69%)	1508 (65%)	934 (69%)	1275 (73%)	NA
ASA V	1 (< 0.1%)	0 (0%)	0 (0%)	1 (< 0.1%)	NA
Unknown	11 (< 0.1%)	6 (< 0.1%)	4 (< 0.1%)	1 (< 0.1%)	NA
Procedure category (*n*, %)					** < 0.001**
Roux-en-Y gastric bypass	598 (11%)	357 (15%)	122 (9.0%)	119 (6.8%)	NA
Sleeve gastrectomy	4672 (86%)	1842 (80%)	1214 (89%)	1616 (92%)	NA
Gastric band placement	68 (1.3%)	55 (2.4%)	7 (0.5%)	6 (0.3%)	NA
Gastric band revision	3 (< 0.1%)	3 (0.1%)	0 (0%)	0 (0%)	NA
Other	91 (1.7%)	54 (2.3%)	19 (1.4%)	18 (1.0%)	NA
Operative time, min (median, IQR)	72 (53, 100)	75 (55, 105)	73 (54, 98)	65 (51, 93)	** < 0.001**

Statistically significant values are marked in bold

Continuous variables reported as median (IQR) with *p* values from Kruskal–Wallis test; categorical variables as count (%) with *p* values from Fisher’s exact test with simulated *p* values

Sleeve gastrectomy was the dominant procedure throughout the study period, increasing from 80% of cases in the pre-COVID period to 92% during recovery, while Roux-en-Y gastric bypass declined from 15 to 6.8% (*p* < 0.001). Gastric band procedures were rare and nearly absent during the COVID and recovery phases (2.5 to 0.3%). Median operative time decreased from 75 min (IQR 55–105) in the pre-COVID period to 65 min (IQR 51–93) during recovery (*p* < 0.001), possibly reflecting the shift toward a higher proportion of sleeve gastrectomy procedures over time (Table 1).

### Racial and ethnic composition of the surgical cohort

**Fig. 2:**
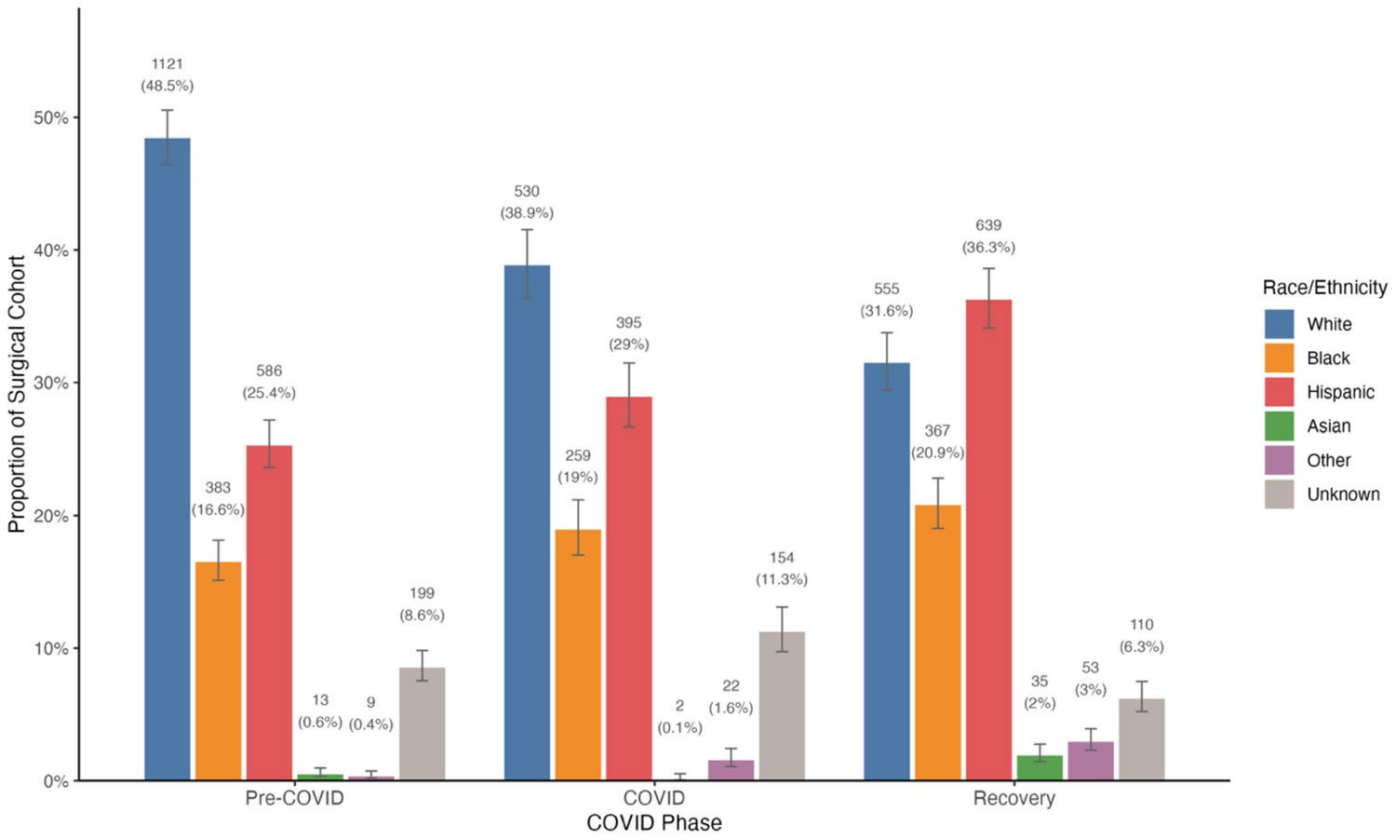
Racial and ethnic composition of the surgical cohort by pandemic phase. Bars represent within-phase proportions; error bars indicate 95% Wilson confidence intervals. Numbers above bars indicate absolute counts and proportions

The racial and ethnic composition of the surgical cohort shifted substantially across pandemic phases (Fig. 2). In the pre-COVID period, White adolescents comprised 49% of the surgical cohort. This proportion declined to 39% during the acute COVID phase and 32% during recovery. Over the same interval, the proportion of Hispanic adolescents increased from 25 to 29% to 36%, and Black adolescents from 17 to 19% to 21%. The overall racial and ethnic composition of the cohort differed significantly across pandemic phases (*p* < 0.001). The proportions of Asian (0.6–2.0%), Other (0.4–3.0%), and Unknown (6.3–11%) patients remained small and did not show consistent directional trends.

To contextualize the observed compositional shifts, we examined annualized procedure volumes within each group. Annualized volumes increased across all racial and ethnic groups over the study period. White patients underwent surgery at approximately 224 procedures per year pre-COVID and approximately 278 per year during recovery—a modest absolute increase. Over the same interval, Hispanic patients increased from approximately 117 procedures per year pre-COVID to approximately 320 per year during recovery, and Black patients from approximately 77 to approximately 184 per year.

### Comorbidity burden by race/ethnicity and COVID phase

Comorbidity burden among White, Black, and Hispanic adolescents across pandemic phases is summarized in Table 2. Black patients consistently had higher median BMI than White and Hispanic patients across all phases (48–50 kg/m^2^ versus 45–46 kg/m^2^ for both other groups), though BMI did not change significantly within any group across phases. Diabetes prevalence was higher among Black (19–22%) and Hispanic (16–19%) patients compared with White patients (13–15%) across all phases. Obstructive sleep apnea rates were broadly similar across all three groups and phases.

**Table 2:** Comorbidity burden by race/ethnicity and pandemic phase among non-Hispanic White, non-Hispanic Black, and Hispanic patients

Group	Characteristic	Pre-COVID *N* = 1,121	COVID *N* = 530	Recovery *N* = 555	*p* value^a^
Non-Hispanic White (*n* = 2206)	BMI (kg/m^2^)	46 (41, 51)	45 (41, 50)	46 (41, 51)	0.8
	Diabetes	151 (13%)	68 (13%)	84 (15%)	0.5
	Obstructive sleep apnea	209 (19%)	102 (19%)	103 (19%)	> 0.9
	Hypertension	41 (5.8%)	21 (4.0%)	32 (5.8%)	0.3
	Hyperlipidemia	15 (1.3%)	17 (3.2%)	18 (3.2%)	
	GERD	165 (15%)	64 (12%)	71 (13%)	0.3
	Active smoker	45 (4.0%)	21 (4.0%)	10 (1.8%)	
	History of prior surgery	12 (1.1%)	1 (0.2%)	4 (0.7%)	
	ASA classification				** < 0.001**
	ASA I–II	404 (36%)	158 (30%)	142 (26%)	
	ASA III–IV	715 (64%)	371 (70%)	413 (74%)	
	ASA V	0 (0%)	0 (0%)	0 (0%)	
	Unknown	2	1	0	
Non-Hispanic Black (*n* = 1009)	BMI (kg/m^2^)	48 (44, 55)	50 (44, 57)	49 (44, 55)	0.2
	Diabetes	81 (21%)	57 (22%)	71 (19%)	0.7
	Obstructive sleep apnea	94 (25%)	69 (27%)	76 (21%)	0.2
	Hypertension	30 (11%)	27 (10%)	21 (5.7%)	0.029
	Hyperlipidemia	9 (2.3%)	5 (1.9%)	4 (1.1%)	
	GERD	54 (14%)	20 (7.7%)	21 (5.7%)	** < 0.001**
	Active smoker	4 (1.0%)	3 (1.2%)	3 (0.8%)	
	History of prior surgery	6 (1.6%)	1 (0.4%)	1 (0.3%)	
	ASA classification				0.095
	ASA I–II	108 (28%)	58 (22%)	80 (22%)	
	ASA III–IV	273 (72%)	201 (78%)	286 (78%)	
	ASA V	0 (0%)	0 (0%)	1 (0.3%)	
	Unknown	2	0	0	
Hispanic (*n* = 1620)	BMI (kg/m^2^)	46 (41, 51)	45 (41, 51)	46 (41, 51)	0.9
	Diabetes	96 (16%)	66 (17%)	119 (19%)	0.6
	Obstructive sleep apnea	145 (25%)	92 (23%)	188 (29%)	0.061
	Hypertension	40 (9.3%)	23 (5.8%)	64 (10%)	0.053
	Hyperlipidemia	17 (2.9%)	17 (4.3%)	33 (5.2%)	
	GERD	40 (6.8%)	32 (8.1%)	57 (8.9%)	0.4
	Active smoker	8 (1.4%)	7 (1.8%)	4 (0.6%)	
	History of prior surgery	9 (1.5%)	1 (0.3%)	1 (0.2%)	
	ASA classification				0.084
	ASA I–II	178 (30%)	142 (36%)	190 (30%)	
	ASA III–IV	406 (70%)	251 (64%)	448 (70%)	
	ASA V	0 (0%)	0 (0%)	0 (0%)	
	Unknown	2	2	1	

Statistically significant values are marked in bold

Asian, Other, and Unknown groups were excluded due to small sample sizes. *P* values reflect within-group comparisons across phases

^a^*p* values not reported for categories with low event rates (prevalence below 5% in ≥ 1 race-phase strata)

Among Black patients, hypertension prevalence declined from 11% in the pre-COVID period (2016–2019) to 5.7% during recovery (*p* = 0.03), and GERD prevalence declined from 14 to 5.7% (*p* < 0.001). Among White patients, ASA III–IV classification increased from 64 to 74% across phases (*p* < 0.001), while ASA distribution did not differ significantly among Black (*p* = 0.095) or Hispanic (*p* = 0.084) patients. No other consistent directional trends in comorbidity burden were observed across pandemic phases within any racial or ethnic group (Table 2).

### Postoperative outcomes and complications

Unadjusted postoperative outcomes by race/ethnicity and COVID phase are summarized in Table 3. Overall complication rates were low throughout the study period across all racial and ethnic groups. Among Black patients, complication rates declined across pandemic phases, from 3.9% pre-COVID to 0.8% during both the acute COVID phase (absolute change − 3.1%; 95% CI − 5.8% to − 1.5%) and recovery (absolute change − 3.1%; 95% CI − 5.7% to − 2.1%; *p* = 0.004). Complication rates did not differ significantly across phases among White patients (COVID vs. pre-COVID: − 0.9%; 95% CI − 1.8% to 0.0%; recovery vs. pre-COVID: − 0.3%; 95% CI − 1.4% to 0.8%; *p* = 0.27) or Hispanic patients (COVID vs. pre-COVID: − 0.7%; 95% CI − 2.1% to 0.5%; recovery vs. pre-COVID: − 0.4%; 95% CI − 1.8% to 0.4%; *p* = 0.55). Among White patients, 30-day readmission rates declined across phases, from 3.8% pre-COVID to 1.9% during the acute COVID phase and 2.0% during recovery (*p* = 0.032). Readmission and reoperation rates did not differ significantly across phases within any other racial or ethnic group. Median length of stay was 1–2 days across all groups and phases.

**Table 3:** Unadjusted length-of-stay (LOS) and 30-day postoperative outcomes by race/ethnicity and pandemic phase

Race/ethnicity	Pre-COVID (Total *n* = 2311)	COVID (Total *n* = 1362)	Recovery (Total *n* = 1759)	*p* value
NH White	*n* = 1121	*n* = 530	*n* = 555	
Complications (*n*, %)	14 (1.2%)	2 (0.4%)	5 (0.9%)	0.27
Readmission (*n*, %)	43 (3.8%)	10 (1.9%)	11 (2%)	0.032
Reoperation (*n*, %)	13 (1.2%)	1 (0.2%)	3 (0.5%)	0.089
LOS, days (median, IQR)	1 (1–2)	1 (1–2)	1 (1–1)	** < 0.001**
NH Black	*n* = 383	*n* = 259	*n* = 367	
Complications (*n*, %)	15 (3.9%)	2 (0.8%)	3 (0.8%)	**0.004**
Readmission (*n*, %)	14 (3.7%)	13 (5%)	12 (3.3%)	0.503
Reoperation (*n*, %)	3 (0.8%)	3 (1.2%)	1 (0.3%)	0.449
LOS, days (median, IQR)	2 (1–2)	1 (1–2)	1 (1–2)	** < 0.001**
Hispanic	*n* = 586	*n* = 395	*n* = 639	
Complications (*n*, %)	7 (1.2%)	2 (0.5%)	5 (0.8%)	0.549
Readmission (*n*, %)	14 (2.4%)	8 (2%)	19 (3%)	0.652
Reoperation (*n*, %)	11 (1.9%)	2 (0.5%)	4 (0.6%)	0.064
LOS, days (median, IQR)	2 (1–2)	1 (1–2)	1 (1–2)	** < 0.001**
Asian	*n* = 13	*n* = 2	*n* = 35	
Complications (*n*, %)	0 (0%)	0 (0%)	0 (0%)	N/A
Readmission (*n*, %)	0 (0%)	0 (0%)	0 (0%)	N/A
Reoperation (*n*, %)	0 (0%)	0 (0%)	0 (0%)	N/A
LOS, days (median, IQR)	2 (2–2)	1 (1–1)	2 (1.5–2)	0.188
Other	*n* = 9	*n* = 22	*n* = 53	
Complications (n, %)	0 (0%)	0 (0%)	0 (0%)	N/A
Readmission (n, %)	0 (0%)	0 (0%)	1 (1.9%)	N/A
Reoperation (n, %)	0 (0%)	0 (0%)	0 (0%)	N/A
LOS, days (median, IQR)	2 (1–3)	1 (1–2)	1 (1–2)	0.241
Unknown	*n* = 199	*n* = 154	*n* = 110	
Complications (*n*, %)	0 (0%)	2 (1.3%)	0 (0%)	N/A
Readmission (*n*, %)	6 (3%)	1 (0.6%)	0 (0%)	0.067
Reoperation (*n*, %)	0 (0%)	0 (0%)	0 (0%)	N/A
LOS, days (median, IQR)	2 (1–2)	1 (1–2)	1 (1–2)	< 0.001

Statistically significant values are marked in bold

Complications reflect a composite of MBSAQIP-defined events

*P* values from Fisher’s exact test (categorical outcomes) and Kruskal–Wallis test (length of stay)

Complication event counts by race/ethnicity and pandemic phase are shown in Supplemental Fig. 1. Most race/ethnicity-phase strata contained fewer than 10 complication events, with zero events observed in several cells, precluding reliable adjusted analysis. Individual complication frequencies by pandemic phase are summarized in Supplemental Table 1. Given the rarity of individual complications in this adolescent cohort, most specific complication types occurred in single-digit numbers across all phases. Sepsis and urinary tract infection demonstrated significant differences across phases (*p* = 0.008 and *p* = 0.017, respectively), but neither remained significant after Benjamini–Hochberg false discovery rate correction (both adjusted *p* > 0.1). Cardiac arrest, cerebrovascular accident, myocardial infarction, and progressive renal insufficiency occurred too infrequently to permit statistical testing. No individual complication type demonstrated a statistically significant difference across phases after correction (Supplemental Table 1).

In a sensitivity analysis using Firth penalized logistic regression, Black race was independently associated with higher odds of complication compared with White patients in the unadjusted interaction model (OR 3.21; 95% CI 1.55–6.71; *p* = 0.002). Hispanic race was not significantly associated with complication risk (OR 0.99; *p* = 0.98). Race × COVID phase interaction terms did not reach statistical significance in either the unadjusted or adjusted models, though the Black × recovery interaction trended toward attenuation (OR 0.30; 95% CI 0.06–1.36; *p* = 0.117). In the fully adjusted model, higher BMI (*p* = 0.036) and diabetes (*p* = 0.033) were independently associated with increased complication risk. Wide confidence intervals throughout reflect the limited event counts available for modeling.

## Discussion

This national analysis of 5432 adolescents undergoing metabolic and bariatric surgery between 2015 and 2023 demonstrates two principal findings. First, the racial and ethnic composition of the surgical cohort shifted substantially across COVID-19 pandemic phases, with increased representation of historically underserved populations—particularly Hispanic and Black patients—during and after the pandemic. Second, unadjusted complication rate disparities observed among Black patients in the pre-COVID period were not seen in later phases. This study represents one of the largest analyses of adolescent bariatric surgery to evaluate racial disparities across the COVID-19 pandemic.

The compositional shifts we observed—with White representation declining from 49 to 32% and Hispanic representation increasing from 25 to 36%—build directly on a well-established literature documenting the persistent underrepresentation of historically underserved adolescents in bariatric surgery [17, 18]. Although MBS is the most effective and durable intervention for severe adolescent obesity, fewer than 0.1% of eligible adolescents receive surgery [7, 8], and Black and Hispanic adolescents undergo surgery at substantially lower rates than White adolescents, despite bearing a disproportionate burden of severe obesity [16–18]. These differences are hypothesized to be driven by systemic barriers, including referral patterns, insurance coverage, geographic access, and provider perceptions of surgical eligibility [12, 17, 19, 40]. Ballinger et al. recently confirmed in a national MBSAQIP analysis that Black and Hispanic adolescents presented with higher BMI and greater comorbidity burden than White peers despite comparable postoperative outcomes—a pattern consistent with delayed access rather than differential surgical risk [16]. Our study extends this work by examining how the COVID-19 pandemic altered these established patterns.

The rise of sleeve gastrectomy from 80 to 92% and decline of RYGB from 15 to 6.8% mirrors well-documented national trends driven by sleeve gastrectomy’s favorable safety profile and shorter operative time [14, 35, 41, 42]. This procedural shift likely contributes to the decrease in median operative time seen across phases. The long-term implications of this procedure shift for adolescent patients—particularly regarding durability of weight loss and revisional surgery rates—remain an important area of investigation [6]. The increase in male patients from 25 to 32% is a notable demographic trend not widely characterized in the adolescent MBS literature [16, 18, 35]. Whether it reflects changing referral practices, evolving societal attitudes, or increased recognition of cardiometabolic risk in male adolescents warrants further study [2, 13, 15].

Our data suggest that longstanding racial and ethnic imbalances began to shift during the pandemic, with greater representation of historically underserved groups in each successive phase, which aligns with other reports of adolescent bariatric surgery utilization during this time period [16, 33]. Based on annualized procedure volumes, this pattern appears consistent with substantial growth in surgical volume among Hispanic and Black adolescents, rather than a reduction in access among White patients. While it remains possible that pandemic-era risk perception led some White families with greater financial flexibility to defer elective surgery [43], the data suggest that observed shifts were driven by a relative expansion in MBS utilization among Hispanic and Black adolescents. These shifts may reflect evolving referral patterns, patient preferences, or institutional prioritization during periods of elective procedure restrictions [30, 31, 40, 44]. Whether this reflects a durable structural change in access or a transient pandemic-related phenomenon remains an important question for future investigation [31].

Several mechanisms may explain the observed increases in representation of historically underserved groups. Rapid telemedicine adoption during the pandemic created new pathways for surgical evaluation that likely reduced barriers related to transportation, geographic distance, and caregiver availability—barriers that disproportionately affect historically underserved populations and lower-income families [45–47]. Prior studies across surgical specialties have demonstrated that telemedicine expanded access to underserved populations during this period [47, 48], and bariatric programs adopting virtual preoperative pathways have reported improved access among historically underserved racial and ethnic populations [47, 49]. Heightened national and institutional focus on health equity may have broadened referral networks [30, 50, 51], while selective cancelation of elective procedures may have prioritized patients with greater disease severity and comorbidity burden [52]. These mechanisms are not mutually exclusive and warrant additional investigation.

These patterns are particularly notable given the broader social context of the pandemic. Since these patients are adolescents, their access to surgical care depends substantially on caregiver engagement throughout the preoperative evaluation, consent, and recovery process [14]. During the pandemic, Hispanic and Black families were disproportionately represented among essential workers in industries such as food processing, agriculture, transportation, healthcare support, and retail—roles that could not be performed remotely and limited flexibility for healthcare navigation and clinic visits [53, 54]. Under traditional in-person care models, these occupational demands might have been expected to further reduce surgical access for these families. Despite these constraints, representation among these groups increased, suggesting that pandemic-era changes in healthcare delivery may have offset barriers associated with caregiver employment demands. This potential interaction between caregiver employment constraints and bariatric surgery access represents an important area for future study.

Despite increased representation, comorbidity burden among Black and Hispanic patients did not change substantially across pandemic phases. Black patients consistently presented with higher median BMI and rates of diabetes and hypertension than White and Hispanic peers across all phases, which is consistent with the existing literature [16, 21]. If pandemic-era changes in access had allowed adolescents from historically underserved racial and ethnic groups to undergo surgery earlier in their disease course, one might expect to see a reduction in comorbidity burden during recovery. The absence of such a trend suggests that while more adolescents from underserved groups underwent surgery, they did not necessarily access care earlier in their disease course; this is consistent with prior literature indicating that structural barriers to timely referral can persist even when overall access improves [55, 56]. These findings reinforce the need for targeted interventions promoting earlier identification and referral of adolescents from historically underserved racial and ethnic populations, not merely an expansion of surgical capacity.

The attenuation of complication rate differences seen across pandemic phases should be interpreted cautiously. Overall complication rates were low at 1.0%, somewhat lower than the 3.5% reported by Ballinger et al., potentially reflecting differences in age range, procedure mix, or complication definitions [16]. This is consistent with the growing body of literature demonstrating the safety of MBS in pediatric populations [2, 6]. With such low event rates, even small absolute differences produce highly variable estimates, and the lack of statistically significant differences in later phases may reflect limited power rather than true equivalence. Sparse complication events precluded formal adjusted analysis, as illustrated in Supplemental Fig. 1. We performed a sensitivity analysis using Firth penalized regression to account for rare event data, which confirmed that Black race was associated with significantly higher complication odds in the pre-COVID period. However, race × COVID phase interaction terms did not reach significance, indicating that the attenuation of disparities in later phases may reflect limited statistical power rather than confirmed resolution of disparities. Thus, we present unadjusted comparisons, which should be considered descriptive and hypothesis-generating.

This study has several important limitations. Its observational design precludes causal inference. MBSAQIP captures only patients who underwent surgery at accredited centers, missing disparities at the referral and evaluation stages where access barriers are likely most prevalent [17, 46]. Participating centers are disproportionately high-volume academic programs, limiting generalizability to community and safety-net settings [35]. The database also lacks socioeconomic variables, including insurance status, income, and geographic access to care, which are central to understanding the mechanisms underlying racial and ethnic disparities in surgical access [17, 28]. Race/ethnicity and socioeconomic status (SES) are closely intertwined in the United States due to structural factors such as wealth inequality, residential segregation, and differential educational opportunity; however, they are not interchangeable. Studies using insurance-stratified analyses have found that racial disparities in bariatric surgery utilization persist even among insured patients, suggesting that race/ethnicity exerts effects beyond those mediated by insurance coverage or income alone [17, 55]. These may include differences in referral patterns, healthcare navigation, perceptions of surgical candidacy, and surgical counseling. Because MBSAQIP does not include income, education, or insurance variables, the relative contributions of SES and race/ethnicity to the patterns observed in this study cannot be determined and remain an important area for future prospective research [28].

Since the Participant Use Files are de-identified and lack center identifiers, we could not determine whether the same institutions contributed data across all pandemic phases or characterize the geographic and socioeconomic profiles of participating centers. If higher-volume academic programs disproportionately continued reporting during the pandemic, while safety-net hospitals reduced participation, the observed compositional shifts could partly reflect changes in the reporting pool rather than changes in patient-level access. This limitation is inherent to registry-based analyses and should be considered when interpreting compositional findings. Additionally, variable coding methodology changed across MBSAQIP data collection years, precluding reliable cross-phase comparison of several operative and outcome variables, such as hypertension, 30-day mortality, procedure type, and surgical approach [57]. Smaller racial categories were combined for analysis due to limited sample sizes, which limited our ability to evaluate disparities within specific subpopulations. Finally, sparse complication events across race/ethnicity and phase strata precluded adjusted regression analysis, limiting our ability to identify independent predictors of outcomes within specific subgroups.

## Conclusions

In summary, the COVID-19 pandemic coincided with meaningful shifts in the racial and ethnic composition of adolescents undergoing MBS, reflecting a greater representation of historically underserved groups. These findings suggest that pandemic-era changes in healthcare delivery may have reduced longstanding access barriers, though the mechanisms remain uncertain. Unadjusted complication rate disparities observed among Black patients in the pre-COVID period were not present in later phases; whether this represents a true attenuation of outcome disparities or reflects insufficient power due to sparse events requires prospective confirmation. Persistent comorbidity burden disparities among patients from historically underserved racial and ethnic groups suggest that timely access to surgery remains an important equity goal. Future studies should evaluate whether these compositional shifts are sustained beyond the pandemic period and identify which programmatic or policy changes most effectively promote equitable access to adolescent metabolic and bariatric surgery.

## Supplementary Information

Below is the link to the electronic supplementary material.

Supplementary file1 (DOCX 151 KB)
