# Supplementary material for: Impact of the COVID-19 pandemic on racial disparities in adolescent bariatric surgery: an MBSAQIP analysis
Source: Surg Endosc. 2026 Jun 16;40(7):6249–61. doi: 10.1007/s00464-026-12955-7 (PMC13369314; doi:10.1007/s00464-026-12955-7)
Supplement: Supplementary file 1 — Supplementary file1 (DOCX 151 KB) [file 464_2026_12955_MOESM1_ESM.docx]

**Supplemental Table 1.** **Individual 30-day complication rates by pandemic phase, reported as event count (%).**

| **Complication (n, %)** | **Pre-COVID** | **COVID** | **Recovery** | **p-value** | **p-value**  **(adj, BH)** |
| --- | --- | --- | --- | --- | --- |
| Anastomotic Leak | 0 (0%) | 2 (0.15%) | 1 (0.06%) | 0.111 | 0.372 |
| Cardiac Arrest | 0 (0%) | 0 (0%) | 0 (0%) | NA | NA |
| Cerebrovascular Accident | 0 (0%) | 0 (0%) | 0 (0%) | NA | NA |
| Deep Incisional SSI | 1 (0.04%) | 1 (0.07%) | 0 (0%) | 0.719 | 0.894 |
| Myocardial Infarction | 0 (0%) | 0 (0%) | 0 (0%) | NA | NA |
| Organ Space SSI | 8 (0.35%) | 2 (0.15%) | 2 (0.11%) | 0.255 | 0.554 |
| Pneumonia | 3 (0.13%) | 2 (0.15%) | 1 (0.06%) | 0.774 | 0.894 |
| Progressive Renal Insufficiency | 0 (0%) | 0 (0%) | 0 (0%) | NA | NA |
| Prolonged Ventilator Use | 1 (0.04%) | 1 (0.07%) | 2 (0.11%) | 0.825 | 0.894 |
| Pulmonary Embolism | 2 (0.09%) | 0 (0%) | 1 (0.06%) | 0.795 | 0.894 |
| Sepsis | 7 (0.3%) | 0 (0%) | 0 (0%) | 0.008 | 0.104 |
| Septic Shock | 1 (0.04%) | 1 (0.07%) | 1 (0.06%) | 1 | 1 |
| Superficial Incisional SSI | 10 (0.43%) | 1 (0.07%) | 3 (0.17%) | 0.115 | 0.372 |
| Thrombosis | 0 (0%) | 0 (0%) | 2 (0.11%) | 0.167 | 0.435 |
| Unplanned Intubation | 3 (0.13%) | 1 (0.07%) | 0 (0%) | 0.369 | 0.685 |
| Urinary Tract Infection | 11 (0.48%) | 0 (0%) | 4 (0.23%) | 0.017 | 0.111 |
| Wound Disruption | 4 (0.17%) | 2 (0.15%) | 1 (0.06%) | 0.629 | 0.894 |

**
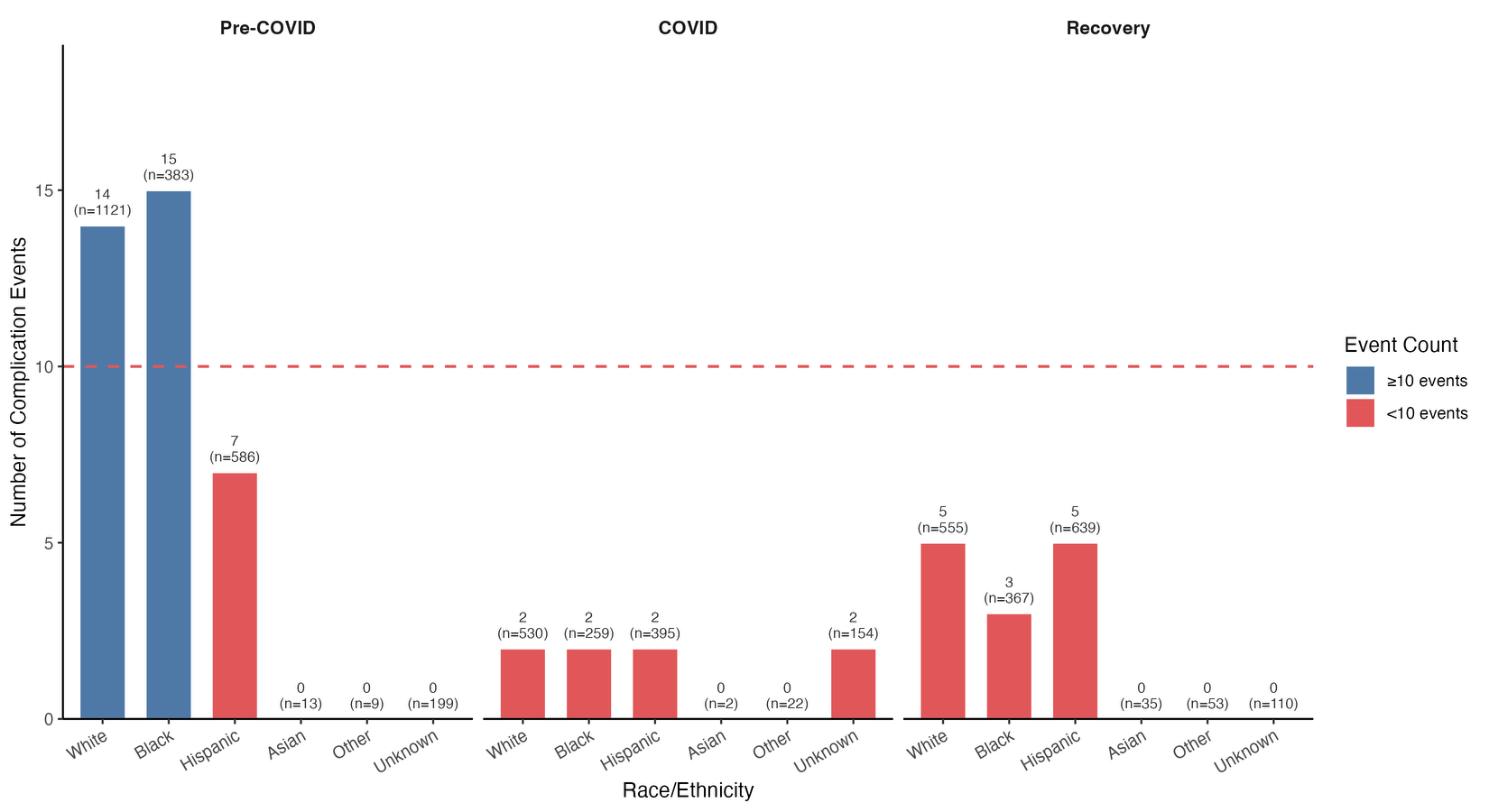
Supplemental Figure 1.** **Complication event counts by race/ethnicity and pandemic phase.**
